# A mysterious connection: arteriovenous fistula between the aortic arch and the left innominate vein

**DOI:** 10.1093/ehjcr/ytad548

**Published:** 2023-11-07

**Authors:** Zakariae Laraichi, Hamza Chraibi, Omar El Aoufir, Nadia Fellat

**Affiliations:** Cardiology A Department, Ibn Sina Hospital, Mohammed V University, Bettouga Street, Rabat 10000, Morocco; Cardiology A Department, Ibn Sina Hospital, Mohammed V University, Bettouga Street, Rabat 10000, Morocco; Emergency Radiology Department, Ibn Sina Hospital, Mohammed V University, Rabat, Morocco; Cardiology A Department, Ibn Sina Hospital, Mohammed V University, Bettouga Street, Rabat 10000, Morocco

## Case description

**Figure 1:**
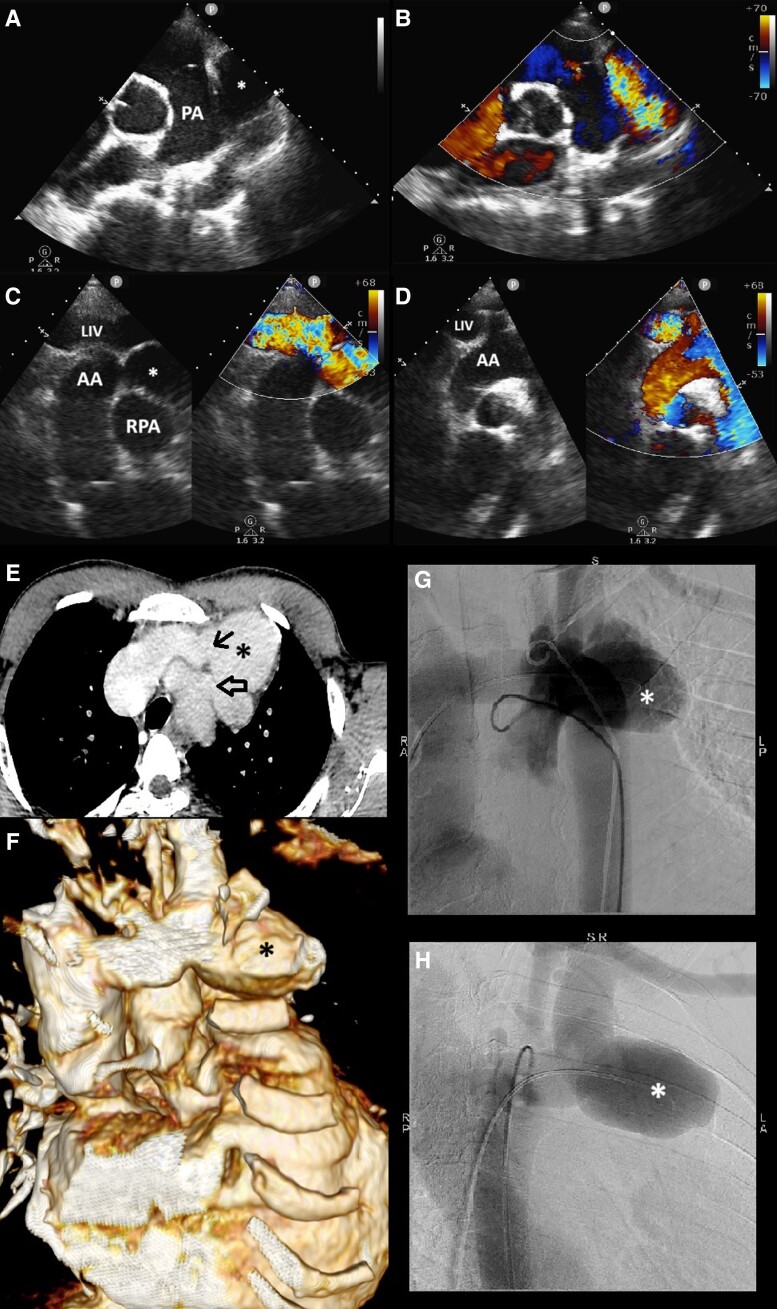
Transthoracic echocardiogram. (*A*) Parasternal short axis view showing a mass next to the pulmonary artery. (*B*) Same view with colour Doppler activated showing intense aliasing. (*C*) and (*D*) Suprasternal view showing the fistula with the left innominate vein. Computed tomography angiography. (*E*) Superior mediastinal axial view showing an arteriovenous communication between the innominate trunk (thin arrow) and the aortic arch (thick arrow). (*F*) 3D reconstruction. Aortic angiography. (*G*) Left anterior oblique view showing a large pseudoaneurysmal structure with a shunt between the aortic arch and the left innominate vein. (*H*) Right anterior oblique view. Star: pseudoaneurysmal vascular structure. AA: aortic arch; LIV: left innominate vein; PA: pulmonary artery; RPA: right pulmonary artery.

A 31-year-old male patient was admitted for a cardiac evaluation. He complained of fatigue and New York Heart Association class II exertional dyspnoea that had started two years previously. He had no personal or family history of trauma or congenital heart disease. Clinical examination revealed pulsatile venous distention in the neck and shoulder areas, hepatomegaly, and a continuous murmur along the midclavicular line to the left of the pulmonary area. Electrocardiography revealed sinus rhythm with negative anterior T waves and right heart strain. Transthoracic echocardiography (TTE) showed that the right atrium was dilated with a surface area of 38 cm² and the right ventricle was enlarged with a basal diameter of 63 mm and a flattened interventricular septum. The main pulmonary artery (PA) was enlarged to 33 mm in diameter. Massive tricuspid regurgitation was present, with a regurgitant area of 65 mm², alongside marked central diastasis and a leaflet coaptation defect of 12 mm. A saccular vascular structure is adjacent to the pulmonary trunk was observed with significant aliasing on TTE colour Doppler, communicating between the aortic wall near its isthmic segment and an extremely enlarged left innominate vein (LIV) (*Figure 1A–D*, see Supplementary material online). Computed tomography (CT) angiography confirmed the presence of a pseudoaneurysmal structure communicating between the aortic arch (AA) and the LIV (*Figure 1E and F*).

Invasive cardiopulmonary catheterization revealed pulmonary hypertension with a severe left-to-right shunt and oxygen saturation of 90% in the superior vena cava and innominate vein. The catheter entered the aneurysm cavity via the retrograde aortic root and the right side. Moderate pulmonary hypertension was observed, with a PA pressure of 45/15/28 mmHg. Aortic angiography confirmed the CT findings (*Figure 1G and H*, see Supplementary material online).

Aortovenous fistulae (AVF) are rare and can be classified as congenital^1,2^ or acquired, mostly as a consequence of trauma or ruptured aortic aneurysm.^3^ Congenital AVF is usually diagnosed at birth or an early age unless the shunt is too small for the patient to present with symptoms.^4^ However, our patient was asymptomatic until the age of 30 years, perhaps indicating that the AVF had grown, and the increasing volume of the shunt exceeded the heart’s compensatory mechanisms, explaining the heart failure symptoms. Surgery remains the treatment of choice for these patients with complex anatomy and a substantial risk of transcatheter occlusion failure. To treat the congestion and symptoms, the patient was prescribed oral furosemide, aldosterone, and bisoprolol. One month after the diagnosis, during the follow-up, the patient reported feeling improved. The patient refused surgery and opted for medical management.

Aortovenous fistulae are a rare diagnosis that should be considered in young patients with shortness of breath and dilated right cavities, especially in post-traumatic contexts. Multimodal and invasive imaging is a valuable tool for assessing this rare condition.

## Supplementary Material

ytad548_Supplementary_Data

## Data Availability

Data sharing is not applicable to this report as no datasets were generated or analysed for this case.
